# More than skin deep: cyclic peptides as wound healing and cytoprotective compounds

**DOI:** 10.3389/fcell.2023.1195600

**Published:** 2023-06-01

**Authors:** Ying-Chiang J. Lee, Bahar Javdan, Alexis Cowan, Keith Smith

**Affiliations:** ^1^ Department of Molecular Biology, Princeton University, Princeton, NJ, United States; ^2^ Rutgers Robert Wood Johnson Medical School, New Brunswick, NJ, United States; ^3^ Department of Microbiology, Perelman School of Medicine, University of Pennsylvania, Philadelphia, PA, United States; ^4^ Merck & Co., Inc., Kenilworth, NJ, United States

**Keywords:** cyclic peptide, wound healing, bioactivity, regeneration, skin, bone, nervous tissue

## Abstract

The prevalence and cost of wounds pose a challenge to patients as well as the healthcare system. Wounds can involve multiple tissue types and, in some cases, become chronic and difficult to treat. Comorbidities may also decrease the rate of tissue regeneration and complicate healing. Currently, treatment relies on optimizing healing factors rather than administering effective targeted therapies. Owing to their enormous diversity in structure and function, peptides are among the most prevalent and biologically important class of compounds and have been investigated for their wound healing bioactivities. A class of these peptides, called cyclic peptides, confer stability and improved pharmacokinetics, and are an ideal source of wound healing therapeutics. This review provides an overview of cyclic peptides that have been shown to promote wound healing in various tissues and in model organisms. In addition, we describe cytoprotective cyclic peptides that mitigate ischemic reperfusion injuries. Advantages and challenges in harnessing the healing potential for cyclic peptides from a clinical perspective are also discussed. Cyclic peptides are a potentially attractive category of wound healing compounds and more research in this field could not only rely on design as mimetics but also encompass de novo approaches as well.

## 1 Introduction

Wounds can generally be grouped into several categories including amputation, avulsion, crush injury, puncture, abrasion, and lacerations. Each wound type can affect multiple tissues—a laceration or puncture may include skin and muscle damage—and could also become chronic wounds. In the United States, chronic skin wounds affect approximately 6.5 million patients, requiring around $25 billion annually on treatment (Sen et al., 2009). Such treatment currently relies on optimizing controllable healing factors rather than administering effective targeted therapies (Liu et al., 2022), (Ezhilarasu et al., 2020). Several approaches for medicated as well as non-medicated dressings have been proposed. Such innovative dressings include hydrogel and alginate materials, electrospun nanofibers, reactive oxygen species scavengers, and microelectronic sensors in smart dressings that aim to facilitate wound healing and provide wound care management (Rezvani Ghomi et al., 2019), (Liu et al., 2021), (Zhao et al., 2020), (Farahani and Shafiee, 2021). Targeted therapies for wound healing that are specific to the extent of damage and tissues affected could add to the current treatment protocols with the potential of restoring both structure and function to patients as well as improving quality of life. Effective therapies may even help to decrease the amount of time patients spend in a healthcare setting, reducing costs for both patients and providers. Innovative wound healing therapeutic and regenerative interventions are much needed, and peptides represent a potential source of lead compound discovery and subsequent development. Owing to their enormous diversity in structure and function, peptides are among the most prevalent and biologically important class of compounds in biology, nature, and modern medicine. Cyclic peptides are polypeptide chains formed by amide bonds in a circular sequence between proteinogenic or non-proteinogenic amino acids but can also occur with disulfide bonds that bring distal residues in a peptide sequence together. One of the most commonly used cyclic peptides in medicine is insulin which has been used clinically for more than 100 years (Bliss, 1993). Currently, cyclic peptides comprise more than two-thirds of the FDA and EMA-approved peptides used in the pharmaceutical industry and approved therapeutic cyclic peptides are derived from natural sources (Zorzi et al., 2017). Cyclization confers many benefits such as promoting peptide stability which improves target protein binding affinity and specificity (Sohrabi et al., 2020); reducing conformational flexibility (Craik, 2006); and increasing peptide chain efficacy by increasing the interacting surface area for protein-protein interactions (Angelini et al., 2012). In addition to improving structural properties, cyclization also improves peptide pharmacokinetics (Bockus et al., 2013). The constrained structure reduces the energy barrier required to bind to transport proteins which increases passive diffusion and active transport (Dougherty et al., 2019). Thus, cyclic peptides are an attractive platform from which wound healing therapeutics could be based. In this review we provide an overview of cyclic peptides that have been shown to promote wound healing as well as recovery and tissue injury prevention. We also discuss the current state of the field clinically, describe applications, and provide an assessment including both advantages and challenges in incorporating cyclic peptides into clinical care. To the best of our knowledge, this is the first review that attempts to collect cyclic peptides from the literature that address wound healing.

## 2 Cyclic wound healing peptides

**FIGURE 1:**
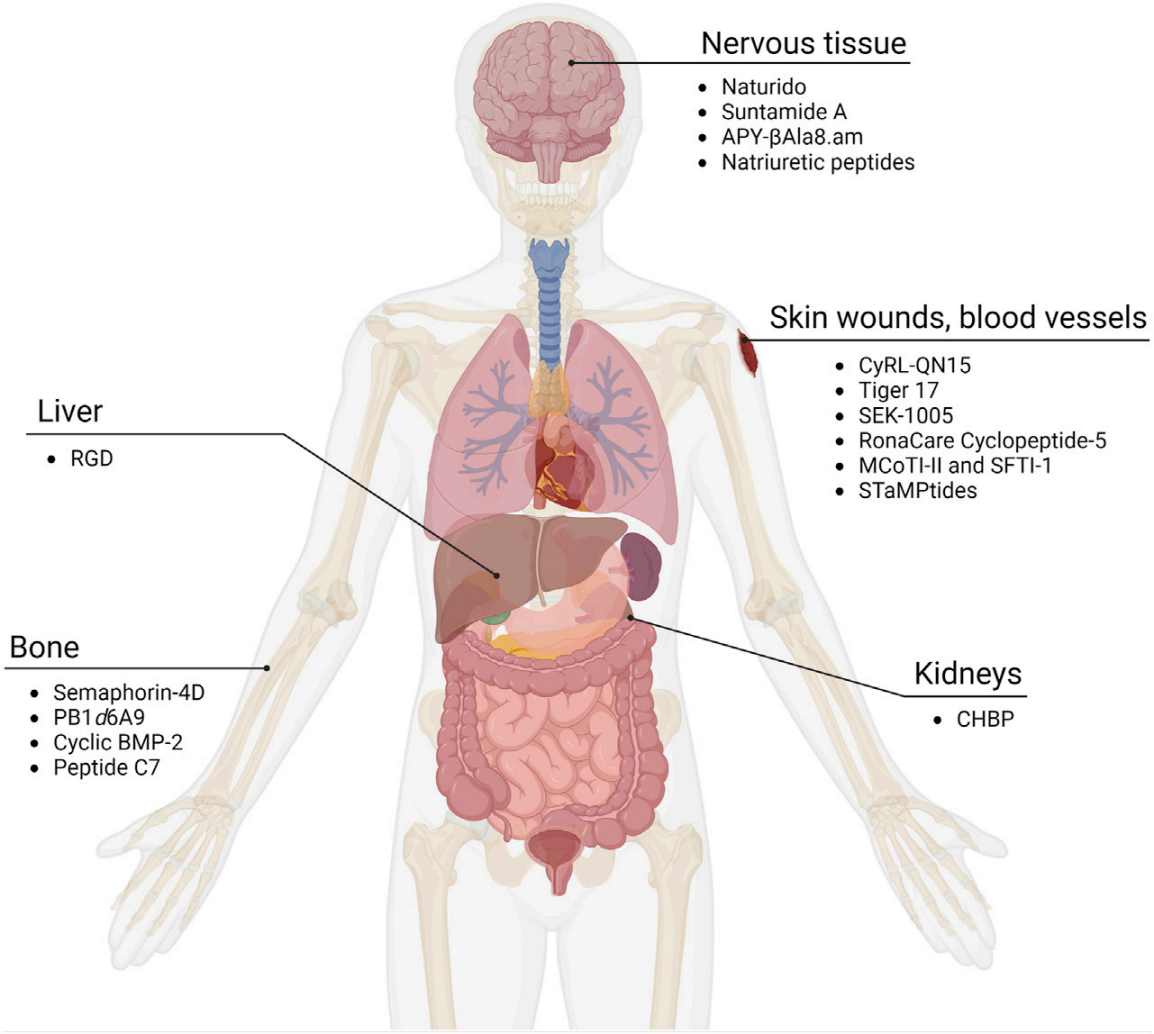
Cyclic peptides as wound healing compounds. Certain cyclic peptides promote growth in tissues while others have been found to reduce damage in organs after ischemia and reperfusion. Adapted from “Human Internal Organs,” by BioRender.com (2023). Retrieved from https://app.biorender.com/biorender-templates.

Injury causing tissue damage can occur throughout the body, and depending on the severity, can involve connective, epithelial, muscle, and nervous tissue. Here, we organize cyclic wound healing peptides into several categories—general wounds of the skin and blood vessels, bone, nervous tissue, and ischemic reperfusion injuries that include the liver and kidneys (Figure 1; Table 1).

**TABLE 1:** Description of cyclic wound healing peptides. Cyclic wound healing peptides described in this review are group according to target tissue type. Details on the experiments and mechanisms are documented.

Target	Cyclic peptide	Mechanism/Function	Model	Ref
General Wound Healing	CyRL-QN15	Influences macrophage activity and promoted regeneration of skin to facilitate wound healing	*In vivo* type 2 diabetes mice; *in vitro*; *ex-vivo* diabetic patient	Fu et al. (2022)
	Tiger 17	Influences the activity of macrophages, keratinocytes, and fibroblasts	*In vitro; in vivo* mouse model full thickness wound	Tang et al. (2014)
	SEK-1005	Induces TGF-β1	*In vivo* mouse model; *in vitro*	Abe et al. (2000)
	RonaCare Cyclopeptide-5	Inhibits ECM protein degradation and promotes ECM stability	n/a	Merck KGaA (2023)
	MCoTI-II	Promotes stability to small proangiogenic peptide fragments	*C. cortunix* egg *in vivo*; *in vitro*	Chan et al. (2011)
	SFTI-1	Promotes stability to small proangiogenic peptide fragments	*C. cortunix* egg *in vivo*; *in vitro*	Chan et al. (2011)
	Single-chain tandem macrocyclic peptides (STaMPtides)	Imitates growth factors and cytokines	*In vitro*	Ito et al. (2022)
Bone	PB1*d*6A9	Inhibits Semaphorin-4D-PlexinB1 interaction via PlexinB1 binding	*In vitro; in vivo* mouse model of postmenopausal osteoporosis	Bashiruddin et al. (2020)
	Peptide C7	Binds with high specificity to bone mesenchymal stem cells (BMSCs)	*In vitro*	Sun et al. (2019)
Nervous System	Naturido	Increases astrocyte proliferation, mRNA expression of NGF and VGF, dendritic length and number, and axon length	*In vitro; in vivo* senescence-accelerated mice (SAMP8)	Ishiguro et al. (2021)
	CATDIKGAEC	Interferes with beta amyloid binding of p75 neurotrophic receptor	*In vitro*	Yaar et al. (2007)
	APY-βAla.8.am	Antagonistically binds to the ephrin binding pocket of EphA4 tyrosine kinase and inhibits EphA4 signaling	*In vitro*	Lamberto et al. (2014)
	Bicyclic and Tricyclic peptide dimers	Mimics part of the BDNF and antagonistically binds to the BDNF receptor trkB	*In vitro*	O’Leary and Hughes (2003)
	Disulfide-rich circular peptides (cyclotides)	Potentially prevents oligomerization of toxic β-amyloid peptides reducing their toxicity	*In vitro; in vivo C. elegans*	Kalmankar et al. (2021)
	Atrial natriuretic peptide (ANP)	Potentially modulates Wnt/β-catenin pathway signaling	*In vitro*	Giovannini et al. (2021)
	Brain natriuretic peptide (BNP)	Potentially modulates Wnt/β-catenin pathway signaling	*In vitro*	Giovannini et al. (2021)
	C-type natriuretic peptide (CNP)	Potentially modulates Wnt/β-catenin pathway signaling	*In vitro*	Giovannini et al. (2021)
	Suntamide A	Promotes activation of transcription factor Nrf2 and consequent antioxidant system induction	*In vitro*	Thapa et al. (2021)
	Bicyclic RGD peptides	Selectively interacts with integrin subunits	*In vitro*	Vedaraman et al. (2021)
Ischemic reperfusion injury (IRI)	IP25	Competitive inhibitor of intracellular adhesion molecular-1 (ICAM-1) dependent that modulates cell adhesion of neutrophils to endothelial tissues	*In vivo* mouse model	Merchant et al. (2003)
	Cyclic peptide RGD	Binds to α5β1 fibronectin integrin receptor and inhibits cell adhesion to fibronectin	*In vivo* rat model of steatotic liver cold ischemia	Fondevila et al. (2009)
	Cyclic helix B peptide (CHBP)	Modulates mTOR1/2	*In vitro, in vivo* mouse model Yang et al. (2014); *in vitro, in vivo* IRI mouse model Yang et al. (2015a); *in vitro* porcine kidney Yang et al. (2015b)	Yang et al. (2014), Yang et al. (2015a), Yang et al. (2015b)

### 2.1 General wound healing: wounds, skin, and blood vessels

The skin, the largest organ in the human body, is the most common surface for formation of wounds, varying in severity based on their level of penetration of the skin layers. While the process of healing depends on wound severity, healing generally begins with the formation of blood clots at the wound site to reduce bleeding. Within a few days, blood vessels in the area are recruited to facilitate delivery of oxygen and nutrients to promote healing. At the same time, the immune system works to fight potential infection at the wound site, as well as aid in repairs. Over the course of weeks, tissue growth and rebuilding of the wound is mediated by red blood cells that work to form collagen and develop granulation tissue over which new skin can form (Ezhilarasu et al., 2020). The timeframe for this multistep process depends strongly not only on the severity of the injury but also on proper wound management.

While cyclic peptides have previously been investigated for use as therapeutics for a range of skin disorders, in this section we focus on the potential of these peptides to increase the rate of wound healing (Namjoshi and Benson, 2010). Recently, a group isolated an optimized cyclic heptapeptide CyRL-QN15 and tested its efficacy in wound healing by loading it onto nanoparticles on a zinc alginate-based hydrogel dressing. This loaded dressing was then applied to full-thickness wounds on type-2 diabetic mice and was found to increase the rate of skin tissue repair at the wound site. Additionally, topical use of the peptide as well as its use as part of the hydrogel was shown to not only reduce inflammation, but also promote epithelization, granulation tissue formation, collagen deposition, and angiogenesis (Fu et al., 2022). A previous study found similar healing benefits when topically applying a small 11 amino-acid peptide, Tiger 17, to a full-thickness wound in a murine model. The healing properties of Tiger 17 were attributed to the peptide’s ability to facilitate macrophage recruitment to the wound site, promote both keratinocyte and fibroblast mediated re-epithelialization and granulation tissue formation, and promote tissue remodeling via release of IL-6 and TGF-ß1— cytokines known to accelerate wound healing (Tang et al., 2014). A novel cyclic peptide, SEK-1005, also demonstrated potent TGF-β1 induction activity in wound healing when topically applied to the dorsal skin of a rat (Abe et al., 2000).

Outside of their direct use promoting faster healing of skin wounds, use of cyclic peptides have been shown to facilitate study of potential wound healing aids, maintain the integrity of the extracellular matrix (ECM), and imitate components involved in the healing process. A study found that formation of a cyclic duplex of collagen mimetic peptides (CMPs) that bind to damaged collagen, which is typically impaired during wound formation, can improve analysis of CMPs as carriers of therapeutic agents that can help wound repair (Ellison et al., 2020). Aside from indirect modulation of ECM stability, a homiletic cyclic peptide known as RonaCare cyclopeptide-5 is shown to directly inhibit degradation of ECM proteins, such as collagenase and elastase (Merck KGaA, 2023). In addition to preventing ECM breakdown, the RonaCare cyclopeptide-5 promotes ECM stability through activation of fibula, laminin, and collagen. While these activities may be utilized to achieve cosmetic goals, including achieving greater skin elasticity and reducing the appearance of wrinkles, increasing stability of the ECM also plays an integral role in promoting wound healing (Xue and Jackson, 2015). Cyclic peptide scaffolds, such as MCoTI-II and SFTI-1, have been shown to provide stability to small peptide fragments with proangiogenic activity, thus indirectly helping to promote angiogenesis which is a critical stage of wound healing (Chan et al., 2011). Similarly, single-chain tandem macrocyclic peptides (STaMPtides) have been shown to mimic growth factors and cytokines in cell culture, allowing them to facilitate wound healing (Ito et al., 2022). The use of cyclic peptides to promote healing, particularly of skin wounds, has promising potential for wound healing therapeutics, although more evidence is needed to support their application in the clinical setting.

### 2.2 Bone healing through modulating osteoblasts and osteoclasts

Injury to bones that result in fractures can take months to heal whereby a multistage process of healing occurs where repair is linked to ossification and remodeling. Recovery from a bone fracture can take months but patient risk factors such as diabetes could complicate the process (Hernandez et al., 2012). A number of compounds have been studied in the context of facilitating bone healing including vitamin C, zinc, the anti-ulcer drug polaprezinc, and peptides (Hart et al., 2015; Pountos et al., 2016; Jiang et al., 2020; O’Connor et al., 2020; Ko et al., 2022). Successful bone healing includes modulating osteoblast and osteoclast activity, and upstream signaling pathways provide ideal targets to increase healing rates. Semaphorin-4D is a molecule that is secreted by osteoclasts and inhibits osteoblast differentiation via the PlexinB1 receptor. The cyclic peptide PB1*d*6A9 was able to inhibit Semaphorin-4D-PlexinB1 interactions and display osteogenic effects both *in vitro* and *in vivo* with an osteoporosis mouse model (Bashiruddin et al., 2020). Other upstream pathways and factors that can be targeted include the bone morphogenetic protein 2 (BMP-2) that display activities in various tissue types. By introducing cysteine residues to cyclize BMP-2, a number of BMP-2 analogues were shown to activate osteogenic activity from the myoblast cell line C2C12 when combined with BMP-2 (Gudivada et al., 2021). Stem cells have also been the target of bone healing compound screens. The peptide C7 was identified using a phage display approach and found to bind to bone mesenchymal stem cells (BMSCs) (Sun et al., 2019). BMSCs grown on a C7-treated biomaterial scaffold increased BMSC adhesion, expansion, and proliferation. While various cyclic peptides have been shown to be effective *in vitro*, more work needs to be done *in vivo* to differentiate compounds that can be applicable to more relevant bone healing situations.

### 2.3 Growth of nervous tissue and associated support cells

Therapeutics that facilitate cellular anabolism and act as protectants are also of interest for their applications to the nervous system. Various cyclic peptides that target various pathways critical for cell regeneration have been shown to help promote the rescue, repair, and regrowth of nerve cells and associated support cells. Naturido, a cyclic peptide consisting of four amino acids isolated from the insect pathogenic fungus *Isaria japonica*, was shown to increase astrocyte proliferation (Ishiguro et al., 2021). Astrocytes are the most abundant type of glial cells that provide a supportive and protective function to neuronal cells in the central nervous system. These cells are also important in neurogenesis. Naturido also increased mRNA expression for nerve growth factor (NGF) and non-acronymic neuropeptide (VGF) in astrocytes. The peptide led to an increase in dendrite length, number, and axon length at certain concentrations. Oral administration of Naturido to senescence accelerated mice (SAMP8) improved spatial learning compared to mice given the cholinesterase inhibitor donepezil that is used to treat symptoms of dementia. NGF homologs have also been found to prevent cell death *in vitro*. The ten amino acid cyclic peptide CATDIKGAEC is derived from NGF and interferes with beta amyloid binding of the p75 neurotrophin receptor (Yaar et al., 2007). This displacement was found to protect neurons from beta amyloid-induced cell toxicity in rat cortical neurons. In addition to p75 neurotrophin receptor inhibition, other studies have targeted receptors involved in modulating neural repair. A modified cyclic peptide, APY-βAla8.am, was designed and bound to the EphA4 receptor tyrosine kinase that has been implicated in retraction of dendritic spines and inhibits axon growth (Lamberto et al., 2014). The peptide antagonist inhibited the collapse of growth cones at the top of axons in nasal retinal explants and was not cytotoxic. Peptide mimetics of natural growth factors have also been explored as possible lead compounds for therapeutics. A bicyclic and tricyclic dimeric peptide were designed to mimic part of the brain-derived neurotrophic factor and acted as agonists of the BDNF receptor trkB (O’Leary and Hughes, 2003). The tricyclic peptide dimer was shown to be effective in embryonic chick sensory neuron survival at picomolar concentrations. In a model of Alzheimer’s Disease in *Caenorhabditis elegans*, disulfide-rich circular peptides from a the butterfly pea plant *Clitoria ternatea* were found to be neuroprotective against beta-amyloid deposits, likely by inhibiting oligomerization (Kalmankar et al., 2021). Other reviews have surveyed cyclic peptides that are growth factor mimetics, such as the above mentioned NGF, specifically for the treatment of Alzheimer’s Disease (Gascon et al., 2021). Neuroprotection in Parkinson’s Disease via cyclic peptides has also been documented. Three types of cyclic natriuretic peptides, atrial natriuretic peptide, brain natriuretic peptide, and C-type natriuretic peptide were protective against *in vitro* examples of studying neurodegeneration seen in Parkinson’s Disease (Giovannini et al., 2021). The Wnt/beta-catenin pathway was implicated in the observed neuroprotective effect. Another peptide, Suntamide A, was found to be neuroprotective via the transcription factor Nrf2 in neuroblastoma cells exposed to oxidative stress (Thapa et al., 2021). While many peptides described here have targeted receptors or intrinsic factors in cells, some cyclic peptides involved in promoting neuronal growth target components of the extracellular matrix (ECM). The ECM consists of collagens, elastin, fibronectin, laminins, tenascins, growth factors, and matrix metalloproteinases (Kular et al., 2014). Cell surface receptors called integrins interact with various ECM components and also participate in signaling. Bicyclic RGD peptides derived from the RGD tripeptide were found to have high affinity for integrin subunits and facilitate nerve growth in a hydrogel application model (Vedaraman et al., 2021). These peptides increased neural cell growth compared to linear or scrambled sequences. The bicyclic RGD peptide treatment also aligned nerve growth when compared to linear RGD and fibronectin controls. Although a number of cyclic peptides have been identified as wound healing and cytoprotective, much remains to be done on how these therapeutics would be administered into patients effectively.

### 2.4 Reducing harm from ischemic reperfusion injury

Cyclic peptides have also been examined in the context of preventing tissue damage, specifically in the context of ischemic reperfusion injury (IRI). Tissues become ischemic when their blood supply is interrupted, as is often seen in strokes and myocardial infarction, and result in cell death. However, when normal blood flow is restored to these tissues in a process called reperfusion, IRIs can occur. Many biological processes have been linked to IRIs including cell death programs, vascular leakage, transcriptional reprogramming, and immune activity (Eltzschig and Eckle, 2011). IRIs can also lead to systemic inflammation and remote organ injury (Naito et al., 2020). Different pharmacological strategies are available as therapies for IRI, and cyclic peptides could be a source of novel agents that prevent or lessen the effects of IRIs. Mice with a hindlimb model of ischemia and reperfusion were treated with the cyclic peptide IP25 and shown to have a 40% reduction in dead tissue if administered without delay during reperfusion (Merchant et al., 2003). IP25 is a competitive inhibitor of the intracellular adhesion molecule-1 dependent cell adhesion between neutrophils and endothelial tissues. The peptide was able to reduce IRI-associated damage by neutrophils and also reduced neutrophil influx. IRIs can be important in the context of organ transplants where the organs are steatotic. Fibronectin production by injured hepatic cells is known and inhibitors of fibronectin cell surface receptors could ameliorate its pathogenic effects in IRI. The cyclic peptide RGD binds to the a5b1 fibronectin receptor and protects against IRI in a liver transplant rat model (Fondevila et al., 2009). Administration of RGD doubled the survival rate of rats post transplant and improved liver function as measured by serum glutamic-oxaloacetic transaminase levels in the transplanted liver. The peptide also decreased macrophage and neutrophil recruitment to the liver, decreased proinflammatory cytokine expression, and inhibited metalloproteinase-9 expression which has been linked to leukocyte migration. Cyclic peptides have also been explored as therapeutics in kidney IRI models. Cyclic helix B peptide (CHBP), derived from the glycoprotein hormone erythropoietin, improved renal function, decreased inflammatory responses, and induced autophagy through modulating mTOR1/2 (Yang et al., 2014). CHBP was also found to be protective in a renal fibrosis mouse IRI model and in isolated porcine kidneys (Yang et al., 2015a; Yang et al., 2015b). IRIs can cause local or distal damage to various tissues and various cyclic peptides have been found to help protect against and mitigate the damage caused by IRIs.

## 3 Wound healing antimicrobial peptides and opportunities to cyclize

A variety of peptides have been characterized that possess both antimicrobial and wound healing properties. In particular, multiple antimicrobial peptides (AMPs) implicated in wound healing have been identified within the epidermis of the skin, which is likely due to its critical function as part of the innate immune system. Thus, as a major interface between the inside and outside world, the epidermis, and, namely, the keratinocytes within the epidermis, play an important role in protecting against infection and facilitating injury remediation. Here, we provide a brief overview of AMPs with dual function as wound healing peptides. While many of these peptides are not cyclic, there are ample opportunities to cyclize and could be subject to peptide engineering efforts.

The linear peptide LL-37, also known as cathelicidin, is one such wound healing AMP. *In vitro*, cathelicidin has been shown to promote the proliferation of multiple cell types including HaCaT cells, a cell line used to model epidermal homeostasis, while also augmenting macrophage recruitment—an important feature of the wound healing process (Wang et al., 2022). Moreover, cathelicidin was shown to boost activity of the wound healing MAPK signaling pathway *in vitro*, and topical cathelicidin was able to accelerate wound healing in *in vivo*, whole skin rodent models of both *S. aureus*-infected and non-infected wounds. Interestingly, CD4-PP—a cyclic derivative of cathelicidin created via its dimerization and backbone cyclization—has been developed and shown to also possess immunomodulatory and antimicrobial activity (White et al., 2022). In particular, CD4-PP has exhibited activity against known pathogens including *E. coli*, *K. pneumoniae*, and *P. aeruginosa.* Furthermore, unlike its linear counterpart, CD4-PP has enhanced resistance to proteolytic degradation which increases its utility in a clinical setting.

Human β defensins are another class of AMP that are associated with wound healing activity. Specifically, human β defensin-3 (hBD-3)—a skin derived AMP—has been shown to boost the migration and proliferation of keratinocytes thereby initiating wound healing (Takahashi et al., 2021). These activities occur via the phosphorylation of the proteins EGFR and STAT, which triggers cascades that stimulate cell migration and proliferation, respectively. Importantly, these effects aren’t limited to the skin alone, with evidence pointing to the efficacy of hBDs in accelerating wound healing in the gut (Otte et al., 2008). In addition to the activities of hBD-3 in keratinocytes, hBD-3 also exerts prominent effects on human fibroblasts as well (Takahashi et al., 2021). Indeed, hBD-3 stimulates the production of pro-angiogenic growth factors including VEGF and FGF, increasing fibroblast proliferation and migration and hastening wound healing in an *in vitro* model. Moreover, *in vivo* models have also demonstrated that hBD-3 enhances immune cell and fibroblast recruitment to skin wound sites as well as angiogenesis. Although very promising therapeutically, there are some barriers that have made human defensins poor therapeutic candidates. For example, their antimicrobial activity is reduced dramatically by ionic strength (Hoover et al., 2003). However, the development of cyclic analogs of the defensins have thus far yielded encouraging results, with some cyclic variants of α defensins exhibiting salt insensitivity (Yu et al., 2000). Additional work in this space is required to understand whether a similar approach may be feasible with β defensins like hBD-3 and if wound healing activity persists.

Catestatin is a neuroendocrine AMP secreted by the chromaffin cells of the adrenal glands. It was first characterized as an inhibitor of catecholamine release and is derived from the proteolytic cleavage of the protein chromogranin A (CgA), which serves as the precursor protein for multiple AMPs. More recent research has revealed pleitropic effects of catestatin, identifying its roles in cardiovascular, metabolic, and immune regulation (Zalewska et al., 2022). In the context of wound healing, it has been shown to stimulate the proliferation and migration of keratinocytes and while also boosting Ca^2+^ levels in these cells (Hoq et al., 2011). These activities were shown to involve multiple signaling pathways including EGFR, Akt, MAPK, and PLC. The active core of catestatin, known as cateslytin, has been shown to be resistant to degradation by *S. aureus*-secreted enzymes and, thus, may have particular relevance in the context of the remediation of infection-prone wounds and growth inhibition of antibiotic-resistant bacteria (Scavello et al., 2021). Additionally, research also suggests that catestatin may also have relevance in the context of inflammatory conditions like ulcerative colitis where it appears to regulate the dynamics of intestinal mucus, reduce severity in an experimental model of colitis, and promote intestinal cell proliferation and migration to facilitate wound healing in the intestinal environment (Eissa et al., 2018). Engineering efforts have been made to cyclize catestatin, however the cyclic derivatives synthesized lost their biological activity, likely due to deviations in the structure of the amino terminal domain (Preece et al., 2004). Therefore, additional efforts are warranted to engineer cyclized forms of catestatin that retain their therapeutic potential.

## 4 Applications and delivery of cyclic wound healing peptides

A number of cyclic peptides have been approved and used in the clinic as antimicrobial, anticancer, and immunosuppressive agents and have a broad range of cellular targets (Choi and Joo, 2020; Zhang and Chen, 2022). The cyclic structure of these agents confers multiple advantages that include an increase in stability and resistance to degradation, specificity, and movement across membranes. Cyclic peptides can also be used in tissue healing applications. The natural process of wound healing can be accelerated with various wound healing agents, and cyclic peptides represent a unique source of potential wound healing therapeutics. In this review, cyclic peptide compounds are presented in the context of not only classic wound healing but also towards preventing reperfusion injuries, anabolism, and regeneration in the context of various tissue types. While typical wound healing can readily be applied to skin traumas such as burns and other open wounds, by broadening our scope we also show that applications to bone and nervous tissue can be accounted for in the context of complex medical traumas. In the context of patient care, a multi-pronged approach to wound healing can be envisioned where cyclic peptides are administered through hydrogels, intravenously, or orally. Multiple cyclic peptides that promote growth of skin, blood vessel, bone, muscular, and nervous tissue could be delivered simultaneously depending on the type and extent of tissue damage in the patient.

Delivery of the cyclic wound healing peptides can be facilitated by nanoparticles and lipid-based systems such as micelles. Peptides used for internal injuries would need to undergo rigorous pharmacokinetic/pharmacodynamic studies to ensure that a therapeutic dose is able to reach the target organ and provide its intended effect without eliciting adverse reactions or clearing from the organ too rapidly. A number of peptides in this review have been for the nervous system and associated support cells, and while drug delivery to the central nervous system is challenging, translocation of compounds across the blood-brain barrier (BBB) can be accomplished using BBB-permeabilizing short peptide tags that could act as delivery vehicles for therapeutic cargo. In addition, cyclic wound healing peptides could be designed and screened to allow for both repair and BBB permeability without the need for adding an additional tag.

Wound healing cyclic peptides and approaches can also be used to accelerate growth of specific tissues in an *ex vivo* setting for therapeutic purposes. Patients who have extensive wounds such as skin trauma may need to receive tissue grafts. Viable patient tissue could be harvested and grown in the laboratory in cell culture supplemented with growth promoting cyclic peptides before being grafted or transplanted back into the patient with the goal of restoring pre-injury functionality and barrier protection. The use of these peptides would help patients recover faster and perhaps even decrease the risk of infection from an open wound.

## 5 Challenges and opportunities for cyclic wound healing peptides

While cyclic peptides are a promising therapeutic intervention for wound healing, there are several limitations preventing their immediate use in clinical settings. There is currently a dearth of randomized control trials evaluating the vast majority of proposed cyclic peptide therapeutics which makes it difficult to determine if they will provide a clinically significant means to improve metrics such as patient mortality or rate of adverse events. As with any therapy, there will be risk of side effects such as allergic reactions to application of these foreign proteins. While immunogenicity is a much greater concern in protein-based therapeutics, that risk is still present for peptides. These challenges are compounded by the increasing use of non-pharmacological interventions in the wound healing space, which may not pose the same degree of risk and are an attractive alternative (Ongarora, 2022). An example of such a technology is Negative Pressure Wound Therapy, which has been shown to improve wound healing times in surgical and traumatic injuries (De Pellegrin et al., 2023; Liebman et al., 2023). Additionally, although studies claim that the cost of these wound healing peptides would be low, currently approved peptide drugs such as man-made insulin often come with significant price tags that are a cause of noncompliance amongst patients (Chiang et al., 2023; Li et al., 2023). Another example is the drug Plecanatide, which was recently approved for treating chronic constipation, and can cost over $500 for a pack (Zhang and Chen, 2022; Plecanatide Prices, 2023). These costs must be considered, especially in the context of the actual benefit achieved in clinical outcomes and not just from laboratory assays. Thus, peptide therapeutics could be limited in use if cost barriers are not properly addressed. It is also important to note that while many of the peptides in this review are categorized as wound healing or cytoprotective for specific tissues, these peptides could also have similar activities in other parts of the body. Such peptides could even be detrimental to the healing process through inducing dysregulation within cells, inflammation, and associated immune responses. More research in relevant models and carefully designed clinical trials are needed to fully characterize bioactivity, safety, and efficacy of these compounds.

Despite these challenges, cyclic peptides are amenable to computational design tools as well as high-throughput functional screening approaches that contribute to lead compound identification and optimization. Computational methods have greatly enhanced our ability to modify existing proteins and design de novo proteins to achieve a precise molecular goal. Software tools such as ProtCAD allow for the intelligent design of de novo proteins with optimized predicted binding energies and stabilities (Summa, 2002; Pike and Nanda, 2015). Various research groups have been able to leverage existing technologies to create cell-permeable cyclic peptides, as well as develop cyclic peptides to specifically target enzyme active sites (Hewitt et al., 2015; Hosseinzadeh et al., 2021). Many of the screening methodologies used for protein identification provide great advantages to therapeutic cyclic peptide discovery programs. One of these advantages is the ability to use phage display to enable rapid, high-throughput screening of many different cyclic peptides at one time. An example of such a platform is the RaPID platform, which can enable the display of even quite complex cyclic peptides with post-translational modifications (Goto and Suga, 2021). RaPID, and other similar peptide display technologies will synergize with traditional peptide therapeutic engineering methods. These optimization methods can help increase oral bioavailability, provide effective cyclization strategies for efficient manufacturing of cyclic peptides, and improve the therapeutic properties of promising peptide lead compounds (Räder et al., 2018; Bechtler and Lamers, 2021).

## 6 Conclusion

Cyclic peptide wound healing, cytoprotectants, and regenerative compounds may help complement current wound treatment approaches and increase the rate of recovery after injury. Here, we show that cyclic peptides can promote healing in various tissue types and even prevent ischemic reperfusion injury. Often, these peptides are derived from host-intrinsic growth factors and target associated receptors. However, developments in computational methods have allowed for de novo protein design and extensive screening approaches could all aid in the design and testing of cyclic peptide wound healing agents. While there are challenges in clinical implementation, cyclic peptides have several native advantages that make it an attractive category of potential wound healing therapeutics.
